# Repeated Use of Prescription Drugs in Pediatrics: Comprehensive Overview Based on German Claims Data

**DOI:** 10.3389/fphar.2021.706682

**Published:** 2021-07-15

**Authors:** Oliver Scholle, Antje Neubert, Oliver Riedel, Irmgard Toni, Ulrike Haug

**Affiliations:** ^1^ Department of Clinical Epidemiology, Leibniz Institute for Prevention Research and Epidemiology—BIPS, Bremen, Germany; ^2^ Department of Paediatrics and Adolescent Medicine, Universitätsklinikum Erlangen, Erlangen, Germany; ^3^ Faculty of Human and Health Sciences, University of Bremen, Bremen, Germany

**Keywords:** adolescents, children, drug utilization study, chronic, pharmacoepidemiology, epidemiology

## Abstract

**Background:** Investigating drug utilization in large and unselected samples of children and adolescents is an important component of public health monitoring. Most existing studies in this field focused on any drug use (i.e., ≥1 prescription of a certain drug) although chronic drug use may be more relevant. This study aimed to provide a comprehensive overview of prevalence and types of prescription drugs used repeatedly in children and adolescents in Germany in 2016.

**Methods:** We used the German Pharmacoepidemiological Research Database (GePaRD)—a claims database covering ∼20% of the German population. We included children and adolescents aged 0–17 years and assessed repeated use of prescription drugs (≥3 prescriptions in 2016) on two levels: therapeutic subgroups (ATC 2nd level) and chemical substances (ATC 5th level). Analyses were stratified by sex and age groups (<2, 2–5, 6–12, and 13–17 years).

**Results:** Overall, 2.5 million children and adolescents were included. In the age groups below 13 years, the prevalence rates of repeated use of prescription drugs (ATC 2nd level) were higher in boys than in girls (113–152 vs. 83–130 per 1,000 person-years), whereas in the age group 13–17 years, they were twice as high in girls than in boys (236 vs. 118 per 1,000 person-years). In boys and girls aged below six years, systemic antibiotics, topical ocular antibiotics, and drugs for constipation were among the most common drugs used repeatedly. For higher ages, methylphenidate, levothyroxine, and combined hormonal contraceptives, were among the most common drugs used repeatedly.

**Conclusions:** Overall, about one in ten children in Germany repeatedly used prescription drugs. This proportion as well as the type of drugs used repeatedly markedly varied by sex and age. For certain drugs, our findings raise concerns regarding appropriateness of prescribing that should be addressed in future studies.

## Introduction

Due to the lack of data from clinical trials, drug therapy in children and adolescents is often associated with uncertainties. Although health authorities in the United States and the EU have implemented policies to facilitate the evaluation of new and old drugs for use in pediatrics, there has not been any noticeable change in the field of clinical research on children and adolescents (European Union, 2006; Elzagallaai et al., 2017; European Commission, 2017). Observational studies on drug therapy in this vulnerable population are therefore indispensable. This includes drug utilization studies (DUS), a valuable tool to quantify and characterize drug exposure of children and adolescents and to identify research priorities regarding the risks and the effectiveness of certain drugs in this population (Neubert et al., 2016).

For DUS on prescription drugs in pediatrics, routinely collected healthcare data offer several advantages over other data sources such as survey data even though healthcare data also have disadvantages (e.g., often limited information on indication, partly more uncertainty as to whether the drug was actually taken). Healthcare data avoid recall and non-responder bias and the typically large sample size in these databases facilitates detailed analyses on certain drugs and subgroups. In addition, these databases provide longitudinal information on drug utilization. Apart from describing any or current use of drugs, it is thus also possible to assess repeated use of drugs. Repeated use of drugs among children and adolescents is an important aspect in many regards: (1) it indicates persistent drug exposure and thus a persistent risk of side effects; (2) it provides information on the burden of chronic disease requiring medical treatment at a young age which is of considerable relevance from the perspective of life-course epidemiology; (3) for certain drugs such as antibiotics, repeated use can indicate inappropriate prescribing requiring preventive regulatory action. However, there has only been a small number of studies investigating repeated drug use in children and adolescents using large healthcare databases. These studies are often based on data from many years ago and do thus not reflect the current situation (Sturkenboom et al., 2008) or focus on specific subpopulations such as children insured by Medicaid (Feinstein et al., 2019). In addition, there are no data at all on repeated use of prescription drugs among children and adolescents in Germany.

We therefore aimed to provide a comprehensive overview on repeated use of prescription drugs in pediatrics based on a large German claims database covering 20% of the German population.

## Materials and Methods

### Data Source

We used the German Pharmacoepidemiological Research Database (GePaRD) for this study (Pigeot and Ahrens, 2008). GePaRD is based on claims data from four statutory health insurance (SHI) providers in Germany and includes information on persons who have been insured with one of the four participating SHI providers since 2004 or later. Per data year, GePaRD covers approximately 20% of the general population of Germany. Prescription data in GePaRD include all reimbursed drugs prescribed by general practitioners and specialists in the outpatient setting. In Germany, about 90% of the general population is covered by SHI providers and there is a free choice of providers. Children are typically covered for free by the SHI policy of one parent or legal guardian and up to the age of 18 years they are also exempt from co-payments, e.g., for prescription drugs. Drugs that are available on prescription only (those evaluated in this study) are reimbursed by SHI providers, with a few exceptions unlikely to be relevant in this study (e.g., so-called lifestyle medications such as those used in erectile dysfunction or male pattern baldness).

### Study Population and Assessment of Drug Prescriptions

We included all children and adolescents aged 0–17 years who fulfilled the following inclusion criterion: continuous insurance coverage throughout the year 2016 (gaps up to 14 days allowed); individuals born or deceased in 2016 were not required to be covered throughout the whole year. Given that only the year of birth is available in GePaRD, age was assessed on December 31, 2016. The year 2016 was the most recent data year available at the time of the analyses.

For each included individual, we identified all prescription drugs dispensed in 2016. Prescriptions were classified based on the German modification of the WHO Anatomical Therapeutic Chemical (ATC) classification system (as per 2018). The German modification fully integrates the system of the WHO, however, adaptations were made, mainly to account for specific aspects of the German drug market, such as the classification of herbal and homeopathic preparations available in Germany only. We excluded prescriptions for vaccines and magistral preparations for this study.

### Data Analysis

We defined repeated use as at least three prescriptions filled on at least three different days in 2016. We assessed repeated use on the level of the therapeutic subgroup (ATC 2nd level) as well as on the level of the chemical substance (ATC 5th level). On the level of the therapeutic subgroup, it was also considered repeated use if, e.g., prescriptions of *different* antibiotics (but all in the same therapeutic subgroup “antibacterials for systemic use” [ATC J01]) were filled on three different days in 2016.

For both ATC levels, we calculated prevalence rates of repeated drug use by dividing the number of persons with repeated use in 2016 by the sum of person-years in the same year. We determined prevalence rates overall and stratified by sex and age groups (<2, 2–5, 6–12, and 13–17 years—following the International Conference on Harmonization guideline (European Medicines Agency, 2001)). Based on these prevalence rates, we determined—for each stratum—(a) the 10 most common therapeutic subgroups used repeatedly (ATC 2nd level) and (b) the 20 most common drugs used repeatedly (ATC 5th level). For comparison, we also calculated the use of any (≥1) of the respective therapeutic subgroups or drugs for each drug and divided the prevalence of repeated use by the prevalence of any use.

In a sensitivity analysis, we varied the definition for repeated drug use (≥2 prescriptions, ≥4 prescriptions). Furthermore, we conducted an analysis on the prevalence of any use of prescription drugs (≥1 prescription). In another sensitivity analysis, we compared the prevalence rates of repeated drug use after excluding “sex hormones and modulators of the genital system” (G03). All statistical analyses were conducted using SAS version 9.4.

## Results

Of 2,798,528 children and adolescents aged 0–17 who had at least one day of insurance coverage in 2016, 2,549,757 (91%) fulfilled the inclusion criterion. Of these, 160,239 were born and 518 died in the study year. In total, the study population accumulated 2,470,692 person-years and there were 4,350,140 prescriptions (Table 1).

**TABLE 1:** Distribution of the study population by sex and age group.

	Total			Girls			Boys		
	N (persons)	N (person-years)	N (prescriptions)	N (persons)	N (person-years)	N (prescriptions)	N (persons)	N (person-years)	N (prescriptions)
Overall Age in years	2,549,757	2,470,692	4,350,140	1,240,059	1,201,381	2,158,914	1,309,698	1,269,311	2,191,226
<2	310,768	231,788	441,068	151,737	113,091	193,329	159,031	118,697	247,739
2–5	561,731	561,700	1,122,794	273,649	273,639	512,882	288,082	288,060	609,912
6–12	937,554	937,525	1,356,536	455,574	455,560	602,311	481,980	481,965	754,225
13–17	739,704	739,679	1,429,742	359,099	359,090	850,392	380,605	380,590	579,350

Note: Prescriptions include drugs available by prescription only.

Identical prescriptions (ATC 5th level) dispensed on the same day were counted only once.

### Prevalence of Repeated Use of Prescription Drugs

**FIGURE 1:**
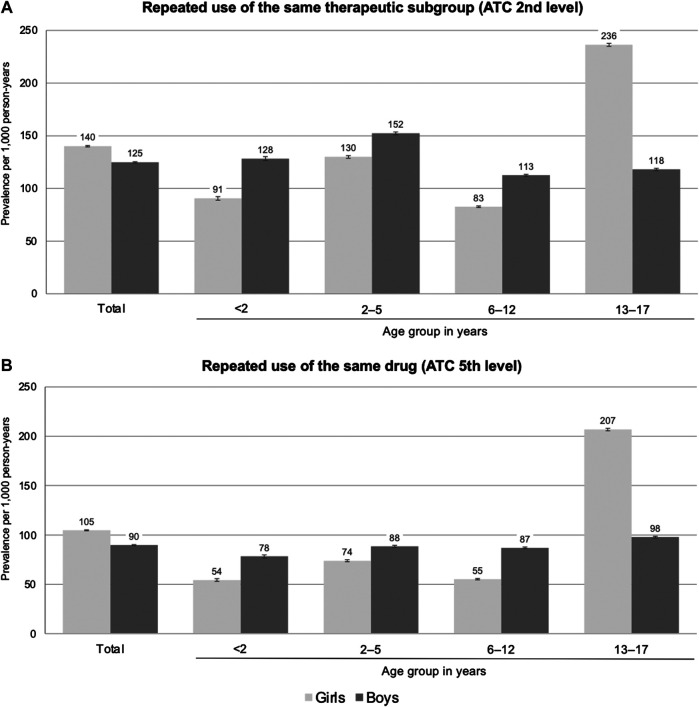
Prevalence of repeated (≥3 per year) use of prescription drugs on the level of therapeutic subgroups **(A)** and on the level of the individual drug **(B)** by sex and age (prevalence per 1,000 person-years; vertical bars indicate 95% confidence intervals).

On the level of therapeutic subgroups, the overall prevalence of repeated use of prescription drugs among children and adolescents was 132 per 1,000 person-years (Supplementary Table S1). In boys, the prevalence of repeated use of prescription drugs was 113–152 per 1,000 person-years in the age groups ≤12 years and 118 per 1,000 person-years in the age group 13–17 years (Figure 1). In girls aged ≤12 years, the prevalence rates were lower than in boys (difference: 22–37 per 1,000 person-years) and showed a peak in the age group 2–5 years. In the age group 13–17 years, the prevalence rates in girls were twice as high as in boys (236 per 1,000 person-years). On the drug level, the patterns by age and sex were similar although the prevalence rates were lower (e.g., the overall prevalence among children and adolescents was 97 per 1,000 person-years; see Supplementary Table S1).

### Most Common Therapeutic Subgroups Used Repeatedly (ATC 2nd Level)

In girls (Table 2), “antibacterials for systemic use” (J01) was among the two most common therapeutic subgroups used repeatedly in all four age groups; in each age group, the prevalence was at least 30 per 1,000 person-years and highest in those aged 2–5 years (66 per 1,000 person-years). In the three youngest age groups, the subgroup “drugs for obstructive airway diseases” (R03) was among the two most common therapeutic subgroups with prevalences of at least 35 per 1,000 person-years (<2 and 2–5 years) and 17 per 1,000 person-years (6–12 years). In the two youngest age groups, the subgroup “ophthalmologicals” (S01) was the third most common subgroup used repeatedly (<2 years: 19 per 1,000 person-years; 2–5 years: 15 per 1,000 person-years) and “drugs for constipation” (A06) was the fourth most common subgroup in those aged 2–5 years (10 per 1,000 person-years). In the oldest age group (13–17 years), “sex hormones and modulators of the genital system” (G03) was the most common therapeutic subgroup used repeatedly with a prevalence of 146 per 1,000 person-years. Also “anti-acne preparations” (D10), “psychoanaleptics” (N06), “drugs for obstructive airway diseases” (R03), and “thyroid therapy” (H03) were among the six most common therapeutic subgroups used repeatedly among girls aged 13–17 years showing all prevalences above 10 per 1,000 person-years.

**TABLE 2:** Top 10 most common therapeutic subgroups used repeatedly in each age group among girls (prevalence per 1,000 person-years).

All girls		<2 years		2–5 years		6–12 years		13–17 years	
Sex hormones and modulators of the genital system (G03)	43.8	Drugs for obstructive airway diseases (R03)	34.9	Antibacterials for systemic use (J01)	66.3	Antibacterials for systemic use (J01)	31.4	Sex hormones and modulators of the genital system (G03)	146.3
Antibacterials for systemic use (J01)	40.6	Antibacterials for systemic use (J01)	32.0	Drugs for obstructive airway diseases (R03)	38.0	Drugs for obstructive airway diseases (R03)	17.4	Antibacterials for systemic use (J01)	35.2
Drugs for obstructive airway diseases (R03)	22.6	Ophthalmologicals (S01)	18.6	Ophthalmologicals (S01)	15.0	Psychoanaleptics (N06)	7.4	Anti-acne preparations (D10)	14.9
Psychoanaleptics (N06)	7.1	Immune sera and immunoglobulins (J06)	5.1	Drugs for constipation (A06)	9.6	Antiepileptics (N03)	3.8	Psychoanaleptics (N06)	14.2
Ophthalmologicals (S01)	7.0	Corticosteroids, dermatological preparations (D07)	3.9	Cough and cold preparations (R05)	7.2	Corticosteroids, dermatological preparations (D07)	2.9	Drugs for obstructive airway diseases (R03)	13.7
Thyroid therapy (H03)	4.8	Vitamins (A11)	3.5	Corticosteroids, dermatological preparations (D07)	4.5	Drugs for constipation (A06)	2.9	Thyroid therapy (H03)	11.6
Anti-acne preparations (D10)	4.7	Corticosteroids for systemic use (H02)	3.1	Corticosteroids for systemic use (H02)	4.2	Thyroid therapy (H03)	2.8	Antiinflammatory and antirheumatic products (M01)	9.2
Corticosteroids, dermatological preparations (D07)	3.7	Cough and cold preparations (R05)	2.5	Antiepileptics (N03)	1.9	Ophthalmologicals (S01)	2.8	Antiepileptics (N03)	4.0
Drugs for constipation (A06)	3.5	Beta blocking agents (C07)	1.9	Anthelmintics (P02)	1.2	Anthelmintics (P02)	2.4	Corticosteroids, dermatological preparations (D07)	3.8
Antiinflammatory and antirheumatic products (M01)	3.3	Drugs for constipation (A06)	1.4	Otologicals (S02)	1.0	Cough and cold preparations (R05)	2.3	Drugs used in diabetes (A10)	3.6

Repeated use was defined as receiving at least three (not necessarily the same kind of) prescription drugs within the same therapeutic subgroup (on different days).

In boys (Table 3), “antibacterials for systemic use” (J01) was among the three most common therapeutic subgroups used repeatedly in all four age groups; in the three younger age groups, the prevalences were higher than in girls and highest in those aged 2–5 years (73 per 1,000 person-years), while in boys aged 13–17 years it was half as high as in girls. Just like for girls, “drugs for obstructive airway diseases” (R03) was among the two most common therapeutic subgroups in the three younger age groups but the prevalences were almost twice as high as in girls. In the two youngest age groups, the subgroup “ophthalmologicals” (S01) was the third most common subgroup used repeatedly (<2 years: 23 per 1,000 person-years; 2–5 years: 18 per 1,000 person-years). In the two highest age groups, “psychoanaleptics” (N06) was among the two most common therapeutic subgroups used repeatedly (6–12 years: 28 per 1,000 person-years; 13–17 years: 34 per 1,000 person-years). In boys aged 13–17 years, “anti-acne preparations” (D10) was the fourth most common therapeutic subgroup used repeatedly, showing a prevalence similar to girls (14 per 1,000 person-years).

**TABLE 3:** Top 10 most common therapeutic subgroups used repeatedly in each age group among boys (prevalence per 1,000 person-years).

All boys		<2 years		2–5 years		6–12 years		13–17 years	
Antibacterials for systemic use (J01)	36.4	Drugs for obstructive airway diseases (R03)	65.3	Antibacterials for systemic use (J01)	72.8	Drugs for obstructive airway diseases (R03)	31.0	Psychoanaleptics (N06)	34.2
Drugs for obstructive airway diseases (R03)	35.7	Antibacterials for systemic use (J01)	42.6	Drugs for obstructive airway diseases (R03)	55.9	Psychoanaleptics (N06)	27.9	Antibacterials for systemic use (J01)	18.8
Psychoanaleptics (N06)	20.9	Ophthalmologicals (S01)	22.8	Ophthalmologicals (S01)	18.0	Antibacterials for systemic use (J01)	26.9	Drugs for obstructive airway diseases (R03)	17.1
Ophthalmologicals (S01)	7.9	Corticosteroids, dermatological preparations (D07)	6.8	Corticosteroids for systemic use (H02)	7.4	Antiepileptics (N03)	4.3	Anti-acne preparations (D10)	14.3
Anti-acne preparations (D10)	4.3	Immune sera and immunoglobulins (J06)	6.3	Cough and cold preparations (R05)	7.4	Nasal preparations (R01)	3.7	Thyroid therapy (H03)	6.3
Corticosteroids, dermatological preparations (D07)	3.9	Corticosteroids for systemic use (H02)	6.1	Drugs for constipation (A06)	7.3	Psycholeptics (N05)	3.5	Antiinflammatory and antirheumatic products (M01)	5.3
Antiepileptics (N03)	3.6	Vitamins (A11)	3.5	Corticosteroids, dermatological preparations (D07)	6.1	Allergens (V01)	3.3	Psycholeptics (N05)	4.5
Corticosteroids for systemic use (H02)	3.4	Cough and cold preparations (R05)	2.8	Antiepileptics (N03)	2.4	Ophthalmologicals (S01)	3.0	Antiepileptics (N03)	4.2
Cough and cold preparations (R05)	3.4	Drugs for constipation (A06)	1.2	Otologicals (S02)	1.3	Corticosteroids, dermatological preparations (D07)	2.9	Drugs used in diabetes (A10)	4.0
Thyroid therapy (H03)	3.0	Antiepileptics (N03)	1.0	Nasal preparations (R01)	1.2	Pituitary and hypothalamic hormones and analogues (H01)	2.8	Nasal preparations (R01)	3.9

Repeated use was defined as receiving at least three (not necessarily the same kind of) prescription drugs within the same therapeutic subgroup (on different days).

The ratio of the prevalence of repeated use to the prevalence of any use was highest for “drugs used in diabetes” (A10)—87 and 91% among 13–17-year-old girls and boys, respectively (Supplementary Tables 2, 3). It was also high for “antiepileptics” (N03) and “psychoanaleptics” (N06). Among 2–5-year-old individuals who were prescribed “antibacterials for systemic use” (J01), the ratio amounted to 17–18%.

### Most Common Drugs Used Repeatedly (ATC 5th Level)

Table 4 and Table 5 show the 20 most common prescription drugs used repeatedly among girls and boys, respectively. Overall, the distribution largely reflects the pattern observed at the level of therapeutic subgroups. Regarding antibacterials for systemic use, the broad-spectrum antibiotic cefaclor showed a similar or higher prevalence as compared to the narrow-spectrum antibiotic amoxicillin in almost all boys and girls aged 12 years or younger. Regarding drugs for obstructive airway diseases, salbutamol preparations showed the highest prevalence. Macrogol was the second most common drug used repeatedly among girls aged 2–5 years (10 per 1,000 person-years) and the fourth most common among boys aged 2–5 years (7 per 1,000 person-years).

**TABLE 4:** Top 20 most common prescription drugs used repeatedly in each age group among girls (prevalence per 1,000 person–years).

All girls		<2 years		2–5 years		6–12 years		13–17 years	
Levonorgestrel and ethinylestradiol (G03AA07)	18.7	Salbutamol, inhalants (R03AC02)	11.1	Salbutamol, inhalants (R03AC02)	14.6	Methylphenidate (N06BA04)	6.3	Levonorgestrel and ethinylestradiol (G03AA07)	62.5
Dienogest and ethinylestradiol (G03AA16)	12.4	Salbutamol, systemic (R03CC02)	8.1	Macrogol, combinations (A06AD65)	9.6	Salbutamol, inhalants (R03AC02)	6.2	Dienogest and ethinylestradiol (G03AA16)	41.6
Salbutamol, inhalants (R03AC02)	8.0	Palivizumab (J06BB16)	5.1	Cefaclor (J01DC04)	7.1	Salmeterol and fluticasone (R03AK06)	3.6	Chlormadinone and ethinylestradiol (G03AA15)	14.8
Methylphenidate (N06BA04)	4.5	Cefaclor (J01DC04)	4.5	Amoxicillin (J01CA04)	6.4	Macrogol, combinations (A06AD65)	2.9	Levothyroxine sodium (H03AA01)	10.5
Levothyroxine sodium (H03AA01)	4.4	Ofloxacin (S01AE01)	4.3	Noscapine (R05DA07)	5.8	Levothyroxine sodium (H03AA01)	2.7	Methylphenidate (N06BA04)	7.1
Chlormadinone and ethinylestradiol (G03AA15)	4.4	Amoxicillin (J01CA04)	4.1	Salbutamol, systemic (R03CC02)	5.1	Cefaclor (J01DC04)	2.4	Ibuprofen (M01AE01)	6.4
Macrogol, combinations (A06AD65)	3.5	Colecalciferol (A11CC05)	3.4	Ofloxacin (S01AE01)	3.5	Amoxicillin (J01CA04)	2.3	Desogestrel and ethinylestradiol (G03AA09)	4.8
Amoxicillin (J01CA04)	3.1	Noscapine (R05DA07)	2.3	Fluticasone (R03BA05)	3.2	Fluticasone (R03BA05)	1.8	Salbutamol, inhalants (R03AC02)	4.4
Cefaclor (J01DC04)	3.1	Propranolol (C07AA05)	1.8	Montelukast (R03DC03)	3.2	Montelukast (R03DC03)	1.5	Desogestrel (G03AC09)	3.9
Salmeterol and fluticasone (R03AK06)	2.7	Gentamicin (S01AA11)	1.7	Budesonide (R03BA02)	2.0	Noscapine (R05DA07)	1.5	Clindamycin and benzoyl peroxide (D10AF54)	3.5
Noscapine (R05DA07)	2.2	Kanamycin (S01AA24)	1.5	Salmeterol and fluticasone (R03AK06)	1.9	Pyrantel (P02CC01)	1.3	Drospirenone and ethinylestradiol (G03AA12)	2.9
Ibuprofen (M01AE01)	2.1	Montelukast (R03DC03)	1.4	Phenoxymethylpenicillin (J01CE02)	1.6	Mometasone (R01AD09)	1.2	Salmeterol and fluticasone (R03AK06)	2.8
Salbutamol, systemic (R03CC02)	2.1	Macrogol, combinations (A06AD65)	1.4	Prednisone (H02AB07)	1.4	Insulin aspart (A10AB05)	1.2	Fluoxetine (N06AB03)	2.6
Fluticasone (R03BA05)	1.7	Ipratropium bromide (R03BB01)	1.3	Ipratropium bromide (R03BB01)	1.4	Budesonide (R03BA02)	1.1	Insulin aspart (A10AB05)	2.2
Montelukast (R03DC03)	1.7	Budesonide (R03BA02)	1.2	Gentamicin (S01AA11)	1.3	Valproic acid (N03AG01)	1.1	Nomegestrol and estradiol (G03AA14)	2.0
Desogestrel and ethinylestradiol (G03AA09)	1.4	Prednisone (H02AB07)	1.1	Methylprednisolone aceponate (D07AC14)	1.3	Phenoxymethylpenicillin (J01CE02)	1.1	Cyproterone and estrogen (G03HB01)	2.0
Ofloxacin (S01AE01)	1.4	Methylprednisolone aceponate (D07AC14)	1.0	Cefuroxime (J01DC02)	1.2	Propiverine (G04BD06)	1.0	Adapalene and benzoyl peroxide (D10AD23)	1.9
Budesonide (R03BA02)	1.2	Fluticasone (R03BA05)	0.9	Cefpodoxime (J01DD13)	1.1	Cefuroxime (J01DC02)	0.9	Mometasone (R01AD09)	1.8
Desogestrel (G03AC09)	1.2	Cefpodoxime (J01DD13)	0.8	Beclometasone (R03BA01)	1.0	Levetiracetam (N03AX14)	0.8	Isotretinoin (D10BA01)	1.8
Insulin aspart (A10AB05)	1.2	Azithromycin (S01AA26)	0.7	Kanamycin (S01AA24)	1.0	Allergen extracts, grass pollen (V01AA02)	0.8	Metamizole sodium (N02BB02)	1.7

Repeated use was defined as receiving at least three (of the same) drugs on different days.

**TABLE 5:** Top 20 most common prescription drugs used repeatedly in each age group among boys (prevalence per 1,000 person-years).

All boys		<2 years		2–5 years		6–12 years		13–17 years	
Methylphenidate (N06BA04)	16.6	Salbutamol, inhalants (R03AC02)	23.3	Salbutamol, inhalants (R03AC02)	22.8	Methylphenidate (N06BA04)	23.4	Methylphenidate (N06BA04)	25.8
Salbutamol, inhalants (R03AC02)	13.3	Salbutamol, systemic (R03CC02)	14.5	Cefaclor (J01DC04)	7.6	Salbutamol, inhalants (R03AC02)	11.0	Salbutamol, inhalants (R03AC02)	5.8
Salmeterol and fluticasone (R03AK06)	4.5	Palivizumab (J06BB16)	6.3	Amoxicillin (J01CA04)	7.6	Salmeterol and fluticasone (R03AK06)	6.7	Levothyroxine sodium (H03AA01)	5.6
Amoxicillin (J01CA04)	3.5	Amoxicillin (J01CA04)	6.1	Macrogol, combinations (A06AD65)	7.3	Fluticasone (R03BA05)	3.6	Lisdexamfetamine (N06BA12)	4.7
Cefaclor (J01DC04)	3.2	Cefaclor (J01DC04)	6.0	Salbutamol, systemic (R03CC02)	6.8	Lisdexamfetamine (N06BA12)	3.6	Salmeterol and fluticasone (R03AK06)	4.1
Salbutamol, systemic (R03CC02)	3.1	Ofloxacin (S01AE01)	5.4	Noscapine (R05DA07)	6.1	Montelukast (R03DC03)	3.0	Ibuprofen (M01AE01)	4.0
Fluticasone (R03BA05)	3.0	Colecalciferol (A11CC05)	3.3	Fluticasone (R03BA05)	5.2	Macrogol, combinations (A06AD65)	2.8	Isotretinoin (D10BA01)	3.7
Macrogol, combinations (A06AD65)	2.9	Montelukast (R03DC03)	2.8	Montelukast (R03DC03)	4.8	Amoxicillin (J01CA04)	2.4	Clindamycin and benzoyl peroxide (D10AF54)	2.9
Montelukast (R03DC03)	2.9	Noscapine (R05DA07)	2.6	Ofloxacin (S01AE01)	4.1	Mometasone (R01AD09)	2.3	Insulin aspart (A10AB05)	2.6
Levothyroxine sodium (H03AA01)	2.8	Budesonide (R03BA02)	2.5	Budesonide (R03BA02)	3.2	Levothyroxine sodium (H03AA01)	2.3	Risperidone (N05AX08)	2.4
Lisdexamfetamine (N06BA12)	2.8	Ipratropium bromide (R03BB01)	2.5	Salmeterol and fluticasone (R03AK06)	2.9	Cefaclor (J01DC04)	2.1	Mometasone (R01AD09)	2.2
Noscapine (R05DA07)	2.4	Prednisone (H02AB07)	2.5	Prednisone (H02AB07)	2.7	Propiverine (G04BD06)	2.0	Atomoxetine (N06BA09)	1.8
Budesonide (R03BA02)	2.0	Fluticasone (R03BA05)	2.3	Ipratropium bromide (R03BB01)	2.2	Budesonide (R03BA02)	2.0	Adapalene and benzoyl peroxide (D10AD23)	1.8
Mometasone (R01AD09)	1.7	Gentamicin (S01AA11)	2.1	Methylprednisolone aceponate (D07AC14)	2.0	Risperidone (N05AX08)	1.7	Valproic acid (N03AG01)	1.5
Ofloxacin (S01AE01)	1.6	Methylprednisolone aceponate (D07AC14)	1.8	Phenoxymethylpenicillin (J01CE02)	1.7	Desmopressin (H01BA02)	1.7	Allergen extracts, grass pollen (V01AA02)	1.5
Risperidone (N05AX08)	1.4	Kanamycin (S01AA24)	1.6	Gentamicin (S01AA11)	1.5	Noscapine (R05DA07)	1.7	Montelukast (R03DC03)	1.3
Ibuprofen (M01AE01)	1.3	Hydrocortisone buteprate (D07AB11)	1.3	Beclometasone (R03BA01)	1.4	Allergen extracts, grass pollen (V01AA02)	1.6	Amoxicillin (J01CA04)	1.1
Insulin aspart (A10AB05)	1.3	Macrogol, combinations (A06AD65)	1.2	Cefuroxime (J01DC02)	1.2	Atomoxetine (N06BA09)	1.6	Somatropin (H01AC01)	1.0
Valproic acid (N03AG01)	1.2	Prednisolone (H02AB06)	1.2	Prednisolone (H02AB06)	1.2	Valproic acid (N03AG01)	1.4	Budesonide (R03BA02)	1.0
Atomoxetine (N06BA09)	1.1	Cefpodoxime (J01DD13)	1.0	Kanamycin (S01AA24)	1.1	Insulin aspart (A10AB05)	1.2	Enoxaparin (B01AB05)	1.0

Repeated use was defined as receiving at least three (of the same) drugs on different days.

In girls aged 13–17 years, the combined contraceptives levonorgestrel and ethinylestradiol (63 per 1,000 person-years) and dienogest and ethinylestradiol (42 per 1,000 person-years in those aged 13–17 years) showed the highest prevalence of repeated use. Combined contraceptives with the following progestogens were also used repeatedly by girls aged 13–17 years (at least 2 per 1,000 person-years): cyproterone, nomegestrol, drospirenone, desogestrel, and chlormadinone. Methylphenidate was the most common drug used repeatedly among girls aged 6–12 years (6 per 1,000 person-years) as well as among boys aged both, 6–12 years (23 per 1,000 person-years) and 13–17 years (26 per 1,000 person-years). In girls aged 6–12 years and 13–17 years as well as in boys aged 13–17 years, levothyroxine was among the five most common drugs used repeatedly.

The ratio of the prevalence of repeated use to the prevalence of any use was highest for the following individual drugs (Supplementary Tables 4, 5): insulin aspart (86–87% in 13–17-year-old girls and boys), levetiracetam (85% in 6–12-year-old girls), valproic acid (84% in 13–17-year-old boys), and methylphenidate (75–79% in 6–12-year-old girls and boys). Among individuals aged 2–5 years, the ratio was 5.6–5.7% for the broad-spectrum antibiotic cefaclor and 4.3–4.8% for the narrow-spectrum antibiotic amoxicillin.

### Sensitivity Analyses

Varying the definition for repeated use on the prevalence estimates (Supplementary Figure 1) showed that with increasing predetermined counts of drug prescriptions (from ≥2 to ≥4 per year), the steepest decline in prevalence was observed from ≥2 to ≥3 prescriptions of the same drug per year (by 52% in girls and 55% in boys). From ≥3 to ≥4 prescriptions, the prevalence decreased by 40% in girls and 43% in boys. Supplementary Figure 1 additionally shows the prevalence of any use (≥1 prescription per year).

After excluding “sex hormones and modulators of the genital system” (G03), the prevalence of repeated use among individuals aged 13–17 years was similar between girls and boys: 120 *vs*. 118 per 1,000 person-years on the level of therapeutic subgroups and 86 *vs*. 98 per 1,000 person-years on the drug level, respectively (Supplementary Figure 2).

## Discussion

The present study provides a comprehensive overview of repeated use of prescription drugs among children and adolescents in Germany. In age groups below 13 years, we found that 11–15% of boys and 8–13% of girls repeatedly received prescription drugs of the same therapeutic subgroup. The types of drugs mainly contributing to these prevalences strongly varied by sex and age. In girls between 13 and 17 years, the prevalence was 24% and thus twice as high as in boys of this age; this difference was mainly driven by oral contraceptives (as shown by the sensitivity analyses). Boys dominated in repeated use of psychoanaleptics (i.e., drugs to treat Attention-Deficit/Hyperactivity Disorder) and drugs for obstructive airway diseases.

Whereas there is a wealth of studies evaluating any drug use (i.e., at least one prescription of a certain drug), there are only few studies investigating repeated drug use—and even fewer studies using a comprehensive approach to investigate repeated drug use, i.e., without restriction to specific drugs, drug classes or patient populations. Sturkenboom et al. (2008) provided an overview of drug use in children and adolescents in the Netherlands, United Kingdom, and Italy based on data from the years 2000–2005. The comparison to our study is hampered, not only because our study using data of 2016 describes drug use more than 10 years later, but also because Sturkenboom et al. assessed the five most commonly used drugs (by anatomical group) for the whole study population only, but not by age and sex. Even though the overall patterns seem to be rather consistent, the prevalences observed by Sturkenboom et al. for the whole study population are the maximum of the range we observed in our study across all age and sex groups, e.g., regarding “antibacterials for systemic use” (all ages: 45 per 1,000 person-years *vs*. 36–41 per 1,000 person-years in our study) and “drugs for obstructive airway diseases” (all ages: 36 per 1,000 person-years *vs*. 23–36 per 1,000 person-years in our study). Also, the comparison with Feinstein et al. (2019) is hampered as their study was conducted in the Medicaid population which has atypical demographic characteristics (Ray and Griffin, 1989). Furthermore, the definition of chronic use was different to our study. In their study, 19% used chronic medication—defined as ≥3 prescriptions for a minimum supply of 30 days each. In our study, the prevalence of repeated use of prescription drugs (also ≥3 prescriptions) was about half as high, and would have been even lower if we had also applied the additional criterion regarding minimum supply. Various reasons may explain this difference, including the fact that over-the-counter (OTC) preparations were not considered in our study and the differences between study populations as mentioned above.

In the following, we present a selection of the most remarkable findings. In most age and sex groups, systemic antibiotics were among the most common therapeutic subgroups used repeatedly. Repeated use of antibiotics has been associated with serious outcomes such as antibiotic resistance, pediatric Crohn’s disease, juvenile idiopathic arthritis, and obesity (Virta et al., 2012; Arvonen et al., 2015; Pouwels et al., 2019; Chelimo et al., 2020). Even though other study designs would be needed to assess appropriateness of antibiotic prescribing (Fleming-Dutra et al., 2016; Smieszek et al., 2018), the high prevalences of repeated prescriptions of antibiotics, and the fact that cefaclor—a broad spectrum antibiotic—was as frequently prescribed as the well-established narrow-spectrum antibiotic amoxicillin, raise concerns regarding the appropriateness of prescribing that should be addressed in future studies. Such studies should also focus on topical ocular antibiotics, particularly the fluoroquinolone ofloxacin, given that prior use of topical fluoroquinolones has been associated with antibiotic resistance (Dave et al., 2011; Fintelmann et al., 2011). Our study showed that these topical drugs are often used repeatedly in children aged 5 years or younger (4–5 per 1,000 person-years).

Contraceptives were the most common repeatedly used prescription drugs in girls aged 13–17 years. While it is not surprising that these drugs are used commonly and repeatedly in this age group, the choice of the drugs raises concerns regarding appropriateness of prescribing. Our analyses at the drug level showed that contraceptives known to have a less favorable benefit-risk balance as compared to other preparations are commonly prescribed. For the combined contraceptives with the progestogens desogestrel, drospiperone, and cyproterone, a higher risk of venous thromboembolism compared to combinations with levonorgestrel has been found (Botzenhardt et al., 2013; Dragoman et al., 2018). For dienogest/ethinylestradiol-containing combined oral contraceptives—the second most common prescription drug used repeatedly in girls in our study—recent research also suggests a higher risk of venous thromboembolism as compared with levonorgestrel/ethinylestradiol-containing preparations (Dinger, 2020). Since 2017, the Federal Institute for Drugs and Medical Devices recommends prescription of oral contraceptives with the lowest risk of venous thromboembolism—primarily those containing levonorgestrel—to minimize the risk of a potential life-threatening drug effect (Federal Institute for Drugs and Medical Devices et al., 2017) (Bundesinstitut für Arzneimittel und Medizinprodukte, BfArM) and Federal Institute for Vaccines and Biomedicines (Paul-Ehrlich-Institut, PEI), 2017).

Macrogol, indicated to treat functional constipation, was also a common drug prescribed repeatedly in both sexes and particularly in the age group 2–5 years. Even though it is known that functional constipation is common in children (Berg et al., 2006; Koppen et al., 2018), we found it surprising that functional constipation requiring medical treatment also shows such a high prevalence. To the best of our knowledge, there is no other study to which we could compare our finding. As functional constipation has been associated with a high disease burden in the pediatric population (Vriesman et al., 2019), future studies about diagnosis and management of this disease in routine care would be of public health interest.

Regarding levothyroxine, there is an ongoing debate about substantial overuse, driven by, e.g., overdiagnosis of subclinical hypothyroidism (Rodriguez-Gutierrez et al., 2017). In Germany, the total number of defined daily doses prescribed between 2006 and 2016 increased by 67% for levothyroxine monotherapy (Ziegler and Schwabe, 2007; Ziegler and Kasperk, 2017). Our study was not designed to assess trends but given that levothyroxine was among the most common drugs in girls and boys aged 13–17 years in our study, such trend studies are needed to assess whether use of levothyroxine has also increased in children and adolescents—which might indicate overuse. A recent international study suggested that an overtreatment of thyroid disease might partly explain the increasing incidence of thyroid cancer in children and adolescents (Vaccarella et al., 2021).

The main strength of our study is the comprehensive assessment of repeated drug use, both at the 2nd and 5th ATC level, in an unselected sample of children and adolescents in Germany using a large database covering 20% of the German population facilitating detailed subgroup analyses. In previous analyses, the data in GePaRD has been shown to be representative regarding drug prescriptions for all persons covered by in Germany (Fassmer and Schink, 2014). We did not include OTC preparations. Even though they are reimbursable for children below 12 years of age, if prescribed by a physician, we assume that they are often dispensed without prescription due to the typically low price, i.e., the data on OTC preparations likely are incomplete in GePaRD. As in any drug utilization study, there is uncertainty whether and to which extent prescribed drugs were actually taken. For repeated drug use, however, we think this uncertainty is lower as compared to any drug use given that regularly filling prescriptions would seem unlikely in the case of complete non-adherence. A further limitation of this study is the lack of direct information on the indication of the drug. In general, linkage of administrative data (as used in this study) with primary data would be useful to fill information gaps, particularly in future studies focusing on a specific disease. However, at least in Germany, linking the data represents a considerable methodological, technical, and data protection challenge, which may explain why there are still relatively few studies linking claims data to other data sources.

While we feel this study was important to provide a general overview on repeated drug use in Germany, and helpful to concretize need for further research, we are fully aware that this general approach does not provide the necessary details to draw definite conclusions for specific conditions or drugs, e.g., with respect to appropriateness of prescribing. Our study should thus be considered as a starting point to be followed by further analyses on specific drugs including further information, e.g., on diagnoses, comorbidity, continuity of treatment, use of diagnostics, and trends in prescribing.

## Conclusion

In conclusion, this study showed that overall, about one in ten children in Germany repeatedly used prescription drugs. This proportion as well as the type of drugs used repeatedly markedly varied by sex and age. For certain drugs (e.g., systemic and topical ocular antibiotics, combined contraceptives), our findings raise concerns regarding appropriateness of prescribing that should be addressed in future studies.

## Data Availability

As we are not the owners of the data, we are not legally entitled to grant access to the data of the German Pharmacoepidemiological Research Database. In accordance with German data protection regulations, access to the data is granted only to BIPS employees on the BIPS premises and in the context of approved research projects. Third parties may only access the data in co-operation with BIPS and after signing an agreement for guest researchers at BIPS. Requests to access the datasets should be directed to Oliver Scholle, scholle@leibniz-bips.de.
