# Supplementary material for: Repeated Use of Prescription Drugs in Pediatrics: Comprehensive Overview Based on German Claims Data
Source: Front Pharmacol. 2021 Jul 15;12:706682. doi: 10.3389/fphar.2021.706682 (PMC8319624; doi:10.3389/fphar.2021.706682)
Supplement: Supplementary file 1 [file DataSheet1.pdf]

Supplementary Material

Supplementary Table 1A. Prevalence of repeated (≥3 per year) use of prescription drugs on the level of therapeutic subgroups (ATC 2nd level) by sex and age

	Prevalence per 1,000 person-years (95% Confidence Interval)		
	**Total**	**Girls**	**Boys**
**Overall**	132.1 (131.7, 132.6)	140.0 (139.4, 140.7)	124.7 (124.1, 125.3)
**Age in years**			
<2	109.9 (108.6, 111.1)	90.5 (88.8, 92.2)	128.3 (126.4, 130.2)
2–5	141.3 (140.4, 142.3)	129.9 (128.6, 131.1)	152.3 (150.9, 153.6)
6–12	98.0 (97.4, 98.6)	82.6 (81.8, 83.4)	112.5 (111.6, 113.4)
13–17	175.4 (174.6, 176.3)	236.3 (234.9, 237.7)	118.1 (117.0, 119.1)

Repeated use was defined as receiving at least three (not necessarily the same kind of) prescription drugs within the same therapeutic subgroup (on different days).

Supplementary Table 1B. Prevalence of repeated (≥3 per year) use of the same prescription drug (ATC 5th level) by sex and age

	Prevalence per 1,000 person-years (95% Confidence Interval)		
	**Total**	**Girls**	**Boys**
**Overall**	97.0 (96.6, 97.4)	104.7 (104.2, 105.2)	89.7 (89.2, 90.2)
**Age in years**			
<2	66.6 (65.6, 67.6)	54.3 (53.0, 55.7)	78.3 (76.7, 79.8)
2–5	81.4 (80.6, 82.1)	73.9 (72.9, 74.9)	88.4 (87.4, 89.5)
6–12	71.6 (71.0, 72.1)	55.2 (54.5, 55.9)	87.0 (86.2, 87.8)
13–17	150.7 (149.9, 151.5)	206.8 (205.5, 208.1)	97.8 (96.8, 98.7)

Repeated use was defined as receiving at least three (of the same) prescription drugs on different days.

Supplementary Table 2. Ratio of the prevalence of repeated use to the prevalence of any use of the 10 most common therapeutic subgroups used repeatedly in each age group among girls

ATC code, individuals with any use (n), individuals with chronic use (n), ratio (%)																			
All girls				<2 years				2–5 years				6–12 years				13–17 years			
G03	88,939	52,610	59.2	R03	25,204	3,945	15.7	J01	106,937	18,150	17.0	J01	116,532	14,319	12.3	G03	81,890	52,521	64.1
J01	343,730	48,716	14.2	J01	27,191	3,614	13.3	R03	52,328	10,402	19.9	R03	38,861	7,919	20.4	J01	93,070	12,633	13.6
R03	140,768	27,193	19.3	S01	28,234	2,104	7.5	S01	62,852	4,094	6.5	N06	4,409	3,387	76.8	D10	27,065	5,362	19.8
N06	12,695	8,506	67.0	J06	1,085	579	53.4	A06	8,954	2,621	29.3	N03	2,144	1,715	80.0	N06	8,251	5,111	61.9
S01	141,619	8,363	5.9	D07	6,805	437	6.4	R05	34,901	1,958	5.6	D07	27,554	1,342	4.9	R03	24,375	4,927	20.2
H03	10,046	5,785	57.6	A11	25,441	398	1.6	D07	20,196	1,231	6.1	A06	5,337	1,341	25.1	H03	7,556	4,176	55.3
D10	30,445	5,626	18.5	H02	9,349	354	3.8	H02	21,538	1,158	5.4	H03	1,993	1,296	65.0	M01	49,868	3,320	6.7
D07	78,591	4,389	5.6	R05	8,309	281	3.4	N03	724	530	73.2	S01	32,422	1,285	4.0	N03	1,906	1,444	75.8
A06	15,495	4,246	27.4	C07	330	213	64.5	P02	6,505	315	4.8	P02	17,754	1,109	6.2	D07	24,036	1,379	5.7
M01	57,744	3,983	6.9	A06	728	154	21.2	S02	7,601	267	3.5	R05	31,820	1,033	3.2	A10	1,503	1,310	87.2

Repeated use was defined as receiving at least three (not necessarily the same kind of) prescription drugs within the same therapeutic subgroup (on different days). The order of the ATC codes is identical to Table 2, which also shows the meaning of the codes.

doi: 10.3389/fphar.2021.706682

Supplementary Table 3. Ratio of the prevalence of repeated use to the prevalence of any use of the 10 most common therapeutic subgroups used repeatedly in each age group among boys

ATC code, individuals with any use (n), individuals with chronic use (n), ratio (%)																			
**All boys**				**<2 years**				**2–5 years**				**6–12 years**				**13–17 years**			
J01	342,017	46,156	13.5	R03	36,252	7,751	21.4	J01	116,884	20,959	17.9	R03	60,871	14,937	24.5	N06	17,742	13,005	73.3
R03	195,849	45,319	23.1	J01	33,249	5,052	15.2	R03	68,849	16,105	23.4	N06	16,286	13,428	82.5	J01	76,978	7,170	9.3
N06	34,128	26,483	77.6	S01	32,992	2,704	8.2	S01	72,620	5,183	7.1	J01	114,906	12,975	11.3	R03	29,877	6,526	21.8
S01	155,056	9,996	6.4	D07	9,704	811	8.4	H02	31,947	2,144	6.7	N03	2,654	2,095	78.9	D10	23,581	5,447	23.1
D10	24,925	5,508	22.1	J06	1,353	748	55.3	R05	36,309	2,118	5.8	R01	19,303	1,792	9.3	H03	4,186	2,404	57.4
D07	88,138	4,957	5.6	H02	14,510	720	5.0	A06	7,086	2,098	29.6	N05	3,460	1,687	48.8	M01	43,504	2,032	4.7
N03	5,840	4,508	77.2	A11	26,098	417	1.6	D07	29,516	1,767	6.0	V01	10,188	1,594	15.6	N05	3,168	1,710	54.0
H02	70,725	4,295	6.1	R05	9,317	336	3.6	N03	892	677	75.9	S01	35,232	1,434	4.1	N03	2,041	1,613	79.0
R05	97,605	4,283	4.4	A06	659	146	22.2	S02	8,969	386	4.3	D07	32,197	1,377	4.3	A10	1,670	1,527	91.4
H03	6,466	3,866	59.8	N03	253	123	48.6	R01	4,568	349	7.6	H01	3,477	1,360	39.1	R01	18,075	1,493	8.3

Repeated use was defined as receiving at least three (not necessarily the same kind of) prescription drugs within the same therapeutic subgroup (on different days). The order of the ATC codes is identical to Table 3, which also shows the meaning of the codes.

doi: 10.3389/fphar.2021.706682

Supplementary Table 4. Ratio of the prevalence of repeated use to the prevalence of any use of the 20 most common prescription drugs used repeatedly in each age group among girls

ATC code, individuals with any use (n), individuals with chronic use (n), ratio (%)																			
All girls				<2 years				2–5 years				6–12 years				13–17 years			
G03AA07	41,194	22,472	54.6	R03AC02	13,107	1,251	9.5	R03AC02	31,143	3,997	12.8	N06BA04	3,849	2,866	74.5	G03AA07	41,112	22,454	54.6
G03AA16	25,462	14,950	58.7	R03CC02	13,432	911	6.8	A06AD65	8,953	2,620	29.3	R03AC02	27,155	2,827	10.4	G03AA16	25,406	14,939	58.8
R03AC02	89,032	9,663	10.9	J06BB16	1,073	579	54.0	J01DC04	34,623	1,931	5.6	R03AK06	4,585	1,656	36.1	G03AA15	9,376	5,306	56.6
N06BA04	7,581	5,418	71.5	J01DC04	9,924	511	5.1	J01CA04	40,597	1,741	4.3	A06AD65	5,336	1,340	25.1	H03AA01	6,773	3,772	55.7
H03AA01	9,158	5,313	58.0	S01AE01	13,376	486	3.6	R05DA07	31,988	1,596	5.0	H03AA01	1,901	1,231	64.8	N06BA04	3,714	2,546	68.6
G03AA15	9,391	5,308	56.5	J01CA04	12,239	461	3.8	R03CC02	18,949	1,403	7.4	J01DC04	27,597	1,075	3.9	M01AE01	45,468	2,290	5.0
A06AD65	15,485	4,240	27.4	A11CC05	25,391	385	1.5	S01AE01	28,500	946	3.3	J01CA04	30,500	1,039	3.4	G03AA09	2,871	1,713	59.7
J01CA04	105,114	3,784	3.6	R05DA07	8,020	260	3.2	R03BA05	3,912	880	22.5	R03BA05	3,559	819	23.0	R03AC02	17,627	1,588	9.0
J01DC04	79,566	3,714	4.7	C07AA05	312	209	67.0	R03DC03	3,669	871	23.7	R03DC03	2,716	667	24.6	G03AC09	3,508	1,409	40.2
R03AK06	9,894	3,209	32.4	S01AA11	7,495	193	2.6	R03BA02	3,797	556	14.6	R05DA07	25,838	662	2.6	D10AF54	8,036	1,248	15.5
R05DA07	74,273	2,627	3.5	S01AA24	6,259	166	2.7	R03AK06	1,554	524	33.7	P02CC01	11,010	587	5.3	G03AA12	1,919	1,028	53.6
M01AE01	52,400	2,507	4.8	R03DC03	893	160	17.9	J01CE02	16,397	425	2.6	R01AD09	7,999	560	7.0	R03AK06	3,612	998	27.6
R03CC02	38,456	2,476	6.4	A06AD65	728	154	21.2	H02AB07	12,242	394	3.2	A10AB05	674	529	78.5	N06AB03	1,700	928	54.6
R03BA05	9,372	2,024	21.6	R03BB01	1,870	146	7.8	R03BB01	3,508	377	10.7	R03BA02	4,120	501	12.2	A10AB05	943	807	85.6
R03DC03	8,730	2,002	22.9	R03BA02	1,126	135	12.0	S01AA11	16,239	368	2.3	N03AG01	607	495	81.5	G03AA14	1,453	715	49.2
G03AA09	2,876	1,715	59.6	H02AB07	5,670	123	2.2	D07AC14	7,538	366	4.9	J01CE02	22,154	489	2.2	G03HB01	1,379	713	51.7
S01AE01	59,797	1,660	2.8	D07AC14	2,427	108	4.4	J01DC02	9,645	339	3.5	G04BD06	1,151	475	41.3	D10AD23	5,852	674	11.5
R03BA02	12,683	1,474	11.6	R03BA05	850	107	12.6	J01DD13	7,140	294	4.1	J01DC02	12,772	411	3.2	R01AD09	10,584	658	6.2
G03AC09	3,522	1,410	40.0	J01DD13	2,329	89	3.8	R03BA01	1,859	287	15.4	N03AX14	440	375	85.2	D10BA01	1,037	630	60.8
A10AB05	1,721	1,400	81.3	S01AA26	3,277	81	2.5	S01AA24	12,549	263	2.1	V01AA02	2,119	344	16.2	N02BB02	15,828	602	3.8

Repeated use was defined as receiving at least three (of the same) prescription drugs on different days. The order of the ATC codes is identical to Table 4, which also shows the meaning of the codes.

doi: 10.3389/fphar.2021.706682

Supplementary Table 5. Ratio of the prevalence of repeated use to the prevalence of any use of the 20 most common prescription drugs used repeatedly in each age group among boys

ATC code, individuals with any use (n), individuals with chronic use (n), ratio (%)																			
**All boys**				**<2 years**				**2–5 years**				**6–12 years**				**13–17 years**			
N06BA04	27,957	21,111	75.5	R03AC02	20,736	2,766	13.3	R03AC02	44,152	6,563	14.9	N06BA04	14,270	11,267	79.0	N06BA04	13,616	9,805	72.0
R03AC02	131,150	16,848	12.8	R03CC02	18,864	1,725	9.1	J01DC04	38,613	2,202	5.7	R03AC02	44,571	5,297	11.9	R03AC02	21,691	2,222	10.2
R03AK06	16,442	5,708	34.7	J06BB16	1,344	747	55.6	J01CA04	45,687	2,198	4.8	R03AK06	8,612	3,250	37.7	H03AA01	3,736	2,149	57.5
J01CA04	112,705	4,465	4.0	J01CA04	15,563	719	4.6	A06AD65	7,086	2,098	29.6	R03BA05	6,589	1,722	26.1	N06BA12	2,297	1,789	77.9
J01DC04	83,461	4,064	4.9	J01DC04	12,287	715	5.8	R03CC02	22,359	1,946	8.7	N06BA12	2,230	1,714	76.9	R03AK06	5,021	1,557	31.0
R03CC02	48,613	3,915	8.1	S01AE01	15,760	637	4.0	R05DA07	33,209	1,760	5.3	R03DC03	4,693	1,448	30.9	M01AE01	40,635	1,517	3.7
R03BA05	16,157	3,814	23.6	A11CC05	26,046	397	1.5	R03BA05	6,282	1,488	23.7	A06AD65	5,122	1,342	26.2	D10BA01	2,831	1,396	49.3
A06AD65	13,325	3,711	27.8	R03DC03	1,722	335	19.5	R03DC03	5,239	1,384	26.4	J01CA04	32,013	1,139	3.6	D10AF54	7,134	1,092	15.3
R03DC03	13,470	3,666	27.2	R05DA07	8,938	312	3.5	S01AE01	32,941	1,176	3.6	R01AD09	13,087	1,116	8.5	A10AB05	1,133	990	87.4
H03AA01	5,897	3,546	60.1	R03BA02	2,127	300	14.1	R03BA02	5,833	929	15.9	H03AA01	1,665	1,090	65.5	N05AX08	1,235	895	72.5
N06BA12	4,534	3,506	77.3	R03BB01	3,107	294	9.5	R03AK06	2,473	828	33.5	J01DC04	26,464	995	3.8	R01AD09	11,580	856	7.4
R05DA07	78,737	2,991	3.8	H02AB07	8,885	291	3.3	H02AB07	18,309	777	4.2	G04BD06	2,054	976	47.5	N06BA09	911	691	75.9
R03BA02	19,631	2,553	13.0	R03BA05	1,722	271	15.7	R03BB01	5,104	624	12.2	R03BA02	7,371	942	12.8	D10AD23	5,330	670	12.6
R01AD09	27,910	2,215	7.9	S01AA11	8,946	255	2.9	D07AC14	8,313	562	6.8	N05AX08	1,206	838	69.5	N03AG01	685	574	83.8
S01AE01	66,087	2,024	3.1	D07AC14	3,416	214	6.3	J01CE02	19,542	480	2.5	H01BA02	2,676	830	31.0	V01AA02	4,472	571	12.8
N05AX08	2,478	1,748	70.5	S01AA24	7,059	188	2.7	S01AA11	19,733	420	2.1	R05DA07	28,459	799	2.8	R03DC03	1,816	499	27.5
M01AE01	47,288	1,665	3.5	D07AB11	2,365	151	6.4	R03BA01	2,845	409	14.4	V01AA02	4,681	775	16.6	J01CA04	19,442	409	2.1
A10AB05	2,017	1,662	82.4	A06AD65	659	145	22.0	J01DC02	10,888	360	3.3	N06BA09	1,019	754	74.0	H01AC01	576	390	67.7
N03AG01	1,802	1,506	83.6	H02AB06	4,857	137	2.8	H02AB06	9,707	341	3.5	N03AG01	780	651	83.5	R03BA02	4,300	382	8.9
N06BA09	1,935	1,448	74.8	J01DD13	2,728	115	4.2	S01AA24	14,255	329	2.3	A10AB05	760	592	77.9	B01AB05	3,001	377	12.6

Repeated use was defined as receiving at least three (of the same) prescription drugs on different days. The order of the ATC codes is identical to Table 5, which also shows the meaning of the codes.

doi: 10.3389/fphar.2021.706682

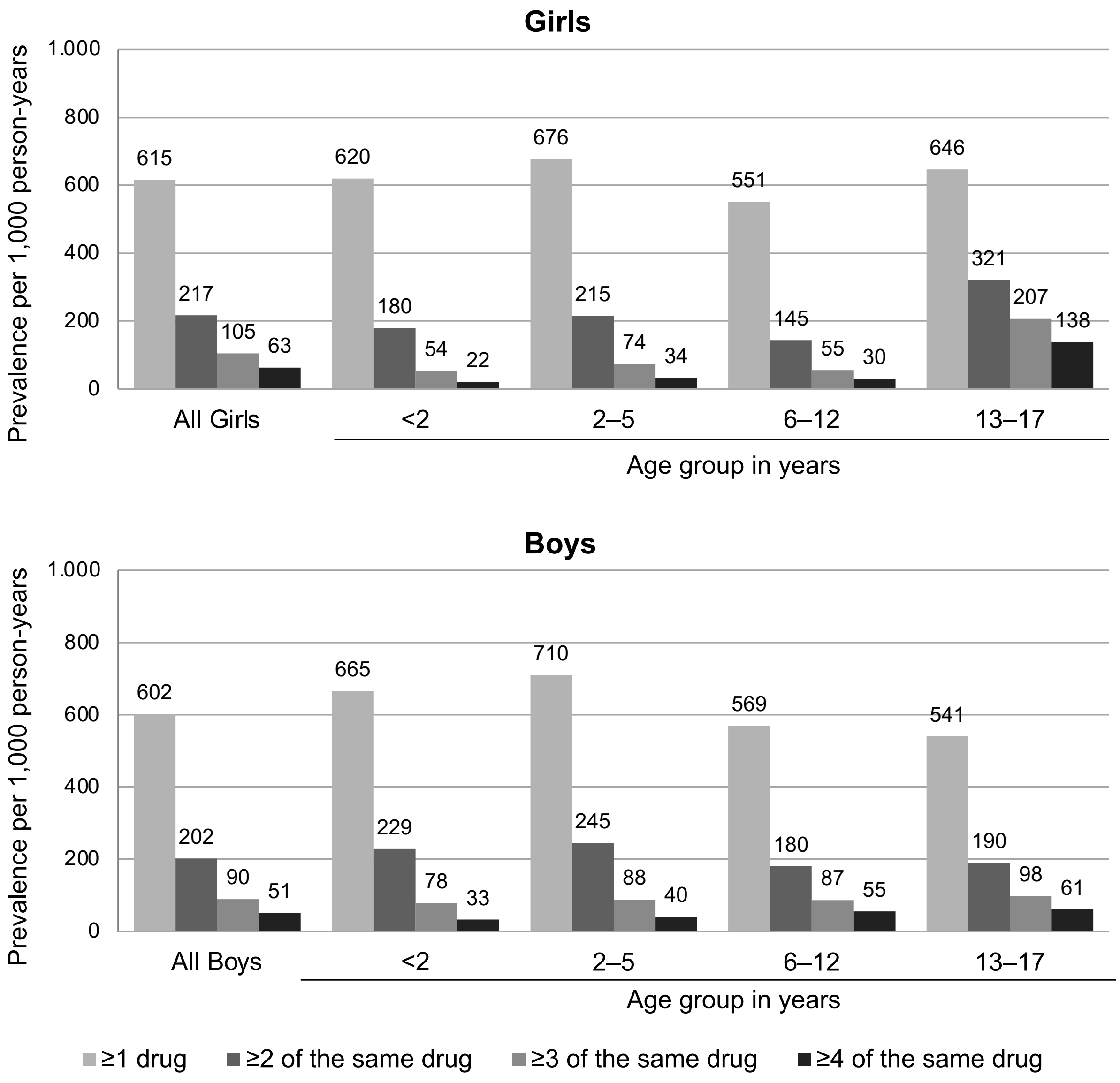

Supplementary Figure 1. Prevalence of any (≥1 per year) and repeated use of prescription drugs based on different counts (per year) by sex and age

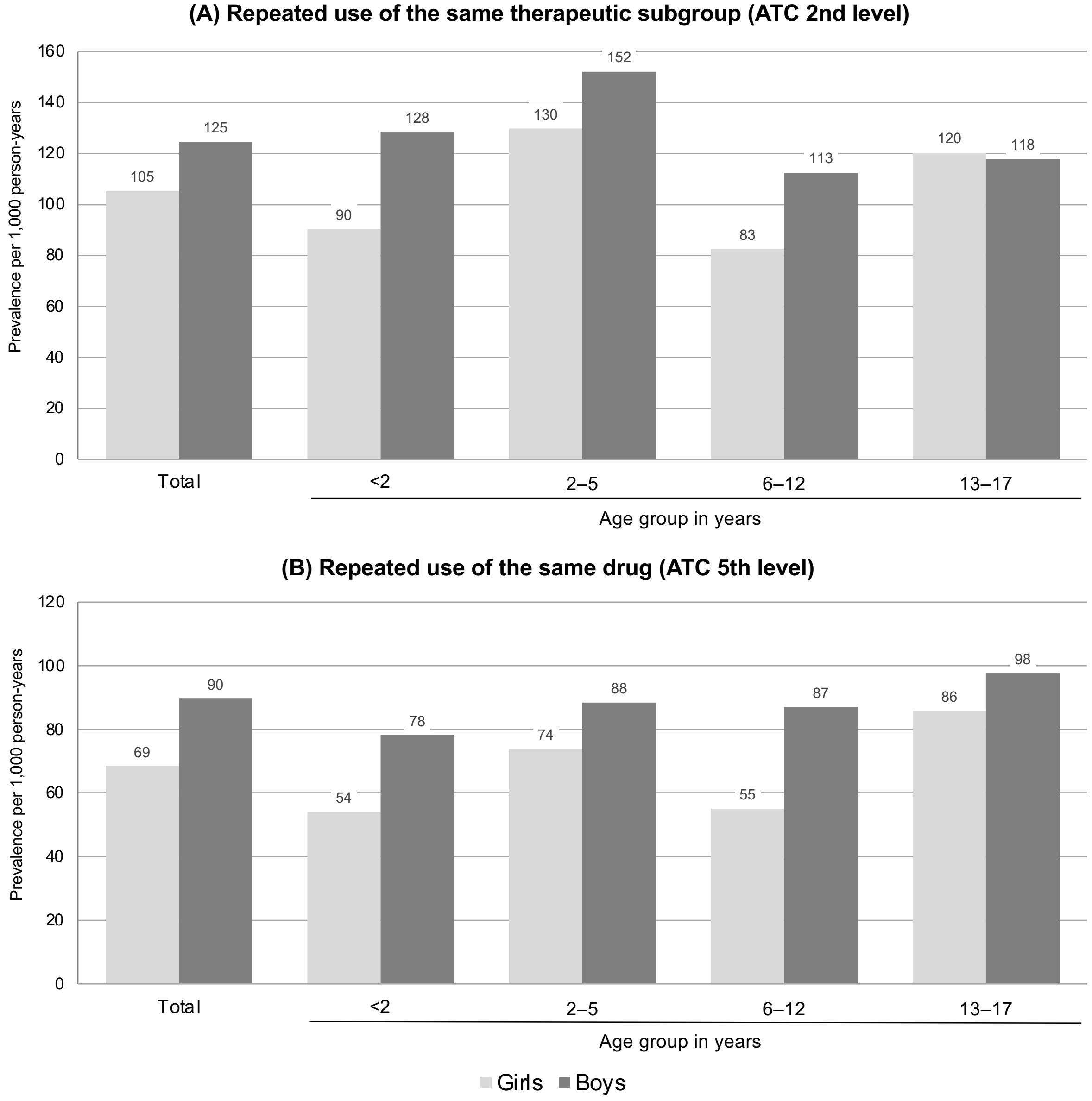

Supplementary Figure 2. Prevalence of repeated (≥3 per year) use of prescription drugs on the level of therapeutic subgroups **(A)** and on the level of the individual drug **(B)** by sex and age after excluding "sex hormones and modulators of the genital system" (ATC G03) (prevalence per 1,000 person-years)
